# The Effect of Aging on the Dynamics of Reactive and Proactive Cognitive Control of Response Interference

**DOI:** 10.3389/fpsyg.2016.01640

**Published:** 2016-11-01

**Authors:** Ling Xiang, Baoqiang Zhang, Baoxi Wang, Jun Jiang, Fenghua Zhang, Zhujing Hu

**Affiliations:** ^1^School of Psychology, Jiangxi Normal UniversityNanchang, China; ^2^Laboratory of Psychology and Cognition Science of Jiangxi, Jiangxi Normal UniversityNanchang, China; ^3^Department of Basic Psychology, School of Psychology, Third Military Medical UniversityChongqing, China

**Keywords:** aging, reactive control, proactive control, activation suppression model

## Abstract

A prime-target interference task was used to investigate the effects of cognitive aging on reactive and proactive control after eliminating frequency confounds and feature repetitions from the cognitive control measures. We used distributional analyses to explore the dynamics of the two control functions by distinguishing the strength of incorrect response capture and the efficiency of suppression control. For reactive control, within-trial conflict control and between-trial conflict adaption were analyzed. The statistical analysis showed that there were no reliable between-trial conflict adaption effects for either young or older adults. For within-trial conflict control, the results revealed that older adults showed larger interference effects on mean RT and mean accuracy. Distributional analyses showed that the decline mainly stemmed from inefficient suppression rather than from stronger incorrect responses. For proactive control, older adults showed comparable proactive conflict resolution to young adults on mean RT and mean accuracy. Distributional analyses showed that older adults were as effective as younger adults in adjusting their responses based on congruency proportion information to minimize automatic response capture and actively suppress the direct response activation. The results suggest that older adults were less proficient at suppressing interference after conflict was detected but can anticipate and prevent inference in response to congruency proportion manipulation. These results challenge earlier views that older adults have selective deficits in proactive control but intact reactive control.

## Introduction

Cognitive control refers to the ability to adjust and optimize inappropriate behavior to achieve a goal-directed action. Previous research has shown that aging affects cognitive control (Hasher et al., 2007; Killikelly and Szűcs, 2013)—when a cognitive task involves a high level of cognitive control, such as an interference task, deficits in cognitive control become evident among older adults (West, 2004; Nessler et al., 2007).

The recent dual mechanisms of cognitive control provide an elegant framework to subtly assess age-related cognitive control decline by distinguishing between proactive and reactive control processes (Botvinick et al., 2001; Braver et al., 2007; Hasher et al., 2007; Braver and West, 2008). The proactive control mode is a form of early selection, in which goal-relevant information is actively maintained before the occurrence of cognitively demanding events to optimally bias attention, perception, and action systems in a goal-driven manner. In contrast, in reactive control, attention is recruited as a late correction mechanism that is mobilized only as needed, in a “just-in-time” manner, such as after a high interference event is detected (Braver et al., 2007; Braver, 2012). While both strategies are equally likely to lead to correct performance on a specific trial, there are some situations in which one or the other would be more appropriate.

In an interference resolution task, two modulations of interference effects have been studied as reflections of cognitive control: sequential congruent (SC) effects and proportion congruent (PC) effects. SC effects refer to the observation that interference effects are typically reduced following incongruent compared to congruent trials (Gratton et al., 1992). In contrast, PC effects refer to the observation that interference effects are typically smaller in contexts with mostly incongruent trials (MI) compared to contexts with mostly congruent (MC) trials (Gratton et al., 1992). In general, SC effects have been considered to reflect reactive control, whereas PC effects are considered to reflect proactive control (Egner, 2007; Egner et al., 2010; Funes et al., 2010; Grandjean et al., 2012; Krug and Carter, 2012; Torres-Quesada et al., 2013). Moreover, one can also distinguish between reactive control applied within the same trial (i.e., the ability to suppress prepotent and distracting or irrelevant information within the same trial) and across trials (i.e., SC effects, or the adjustment of conflict resolution based on a previously experienced conflict level) (Bélanger et al., 2010; Wylie et al., 2010; Puccioni and Vallesi, 2012).

The dissociation of two qualitatively different control mechanisms prompted reconsideration of the ubiquity of age-related declines in interference control. Previous studies have revealed that older adults rely predominantly on reactive control and that they have a selective age-related impairment in proactive control (Botvinick et al., 2001; Braver et al., 2005; Braver and West, 2008; Paxton et al., 2008). It should be noted, however, that these findings were based mainly on the AX-CPT task. It is therefore important to determine whether the pattern of intact reactive control and selective deficits in proactive control observed in older adults is present in tasks other than the AX-CPT, such as commonly employed conflict interference tasks.

Only a few recent studies have employed an interference task to investigate age-related deficits in cognitive control by dissociating proactive and reactive control (Mutter et al., 2005; Bélanger et al., 2010; Czernochowski et al., 2010; Puccioni and Vallesi, 2012). Bélanger et al. (2010) investigated inhibition and goal maintenance in persons with Alzheimer’s disease, mild cognitive impairment, healthy older adults, and younger adults by manipulating the proportion of congruent items in the Stroop task. The results showed that older adults were more sensitive to interference, as evidenced by a disproportionate effect of the congruency context, while goal maintenance was only partially impaired with age. Specifically, older adults needed more time to implement goal-appropriate strategies but did not commit significantly more errors compared to their young counterparts. Puccioni and Vallesi (2012) investigated the effects of cognitive aging on conflict resolution and conflict adaptation. A color–word Stroop task with no feature repetitions was administered to 23 older adults and 22 younger controls. The results showed that the Stroop effect was larger in older adults than in younger controls, suggesting that aging affects a specific process of conflict resolution. These results also confirmed the presence of some conflict adaptation effects that were spared by aging. Mutter et al. (2005) investigated the impact of age and task context on Stroop task performance. The findings showed that older adults were able to evaluate Stroop task demands and modify their representations of task context in response to this knowledge.

However, in previous related studies (Mutter et al., 2005; Bélanger et al., 2010; Czernochowski et al., 2010; except for Puccioni and Vallesi, 2012), the control process might be contaminated by bottom-up associative learning. Specifically, the reduction of the interference effect reflected by SC and PC effects was normally explained by top-bottom strategic control (Lindsay and Jacoby, 1994; Braver et al., 2007). However, it was also explained by pure memory/learning-based processes such as frequency account (Hutchison, 2011; Atalay and Misirlisoy, 2012) or feature repetitions (Mayr et al., 2003; Schmidt and Besner, 2008). In the present study, we sought to reduce the frequency confounds and feature repetitions from the cognitive control measures as much as possible. A prime-target number category task was used in which many prime-target number combinations can be selected. A specific prime-target digit pair was set to repeat with equal frequency rather than different frequencies under both the MC and MI contexts. At the same time, there were no stimulus repetitions between two subsequent trials (for details, see the experimental materials).

Moreover, we used distribution analysis to investigate the dynamics of interference control, using the activation-suppression model of Ridderinkhof (2002a) as a guide for this analysis. The activation-suppression model provides a powerful framework to investigate group differences—namely, by dissociating an early automatic response capture induced by automatic processing from a later controlled top-down response suppression mechanism engaged to reduce the interference from the incorrect response capture. This dynamic cannot be revealed by mean interference effects such as those reported in the previous studies. Indeed, a few recent studies have begun to use distributional analyses to investigate the dynamics of interference control in older adults (Wylie et al., 2010; Ridderinkhof and Wijnen, 2011; Joyce et al., 2014).

Working within the framework of the activation-suppression model, we investigate how aging affects the strength of response capture and the proficiency of suppression in the context of both reactive control and proactive control. With this aim, two groups (older and young adults) performed the task in which the proportion of congruency was manipulated. Following previous literature, we distinguished between two control implementation processes: between-trial reactive control mechanisms, as measured by the reduction of interference effects following an incongruent trial (i.e., SC effects), and proactive control mechanisms, as measured by the reduction of interference effects in the context of MI trials (i.e., PC effects). Moreover, we studied the within-trial reactive control mechanism, which is understood as the activation and suppression of irrelevant information within the same trial, and is measured by larger interference effects.

In summary, in our study, cognitive control was assessed via a relatively “pure” measure for which the performance pattern cannot be explained by pure memory/learning-based processes such as item frequency or stimulus repetition. Furthermore, we used distributional analyses to distinguish between the strength of response capture and the efficiency of suppression in the context of two cognitive control strategies.

## Materials and Methods

### Participants

Thirty undergraduate students were recruited from Jiangxi Normal University (15 men and 15 women; age range: 18–24 years, mean age: 21.5), who received course credit for their participation. Thirty older adults (14 men and 16 women, age range: 65–75 years, mean age: 70.13) were recruited from the communities around the university. The older adults were assessed with the Mini-Mental State Examination (MMSE) and found to have normal general cognitive function (MMSE ≥ 26) (Zhang, 1998). This study was approved and performed in accordance with the guidelines of the ethics committees of Jiangxi Normal University. Written informed consent was obtained from all participants. Participants with a history of neurological psychiatric or vascular disease and who use any psychotropic or hypertensive medications were excluded.

The education level of both groups was matched and both groups had completed at least 12 years of education. There was no significant difference in scores on the Geriatric Depression Scale between younger adults (5.27 ± 2.59) and older adults [6.33 ± 2.47; *F*(1,58) = 2.67, *p* = 0.11; Yesavage et al., 1983]. Furthermore, none of the participants had depression (all had scores below 10; Lopez et al., 2010).

### Materials and Procedure

A prime-target interference task was used for this experiment. The prime and target consisted of the numbers from 1 to 9 except 5. Subjects were asked to press a response key with one hand if the target was larger than 5, and with the other hand if the target was smaller than 5. The assignment of right and left hand keys was counter balanced across subjects. The prime digits were mapped onto the same (i.e., congruent) or an alternative response (i.e., incongruent) as the target. Each trial consisted of a fixation cross (presented for 400–600 ms), a blank (70 ms), a prime (35 ms), a blank (70 ms), and a target (3000 ms). The target was presented until response onset or for a maximum of 3000 ms. Each trial was followed by a 700 ms blank screen. A blank screen was presented in each trial during the inter-trial interval.

Before the formal experiment, participants were required to perform a practice block to familiarize themselves with the experimental procedure and the stimulus-response mapping. In the practice block, the proportion of congruency was 50%. In the formal experimental task, the proportion of congruency was manipulated using two different contexts: the MC context and the MI context.

In the prime-target number category task, the prime and target consisted of numbers from 1 to 9 except 5; there were then 56 prime-target number combinations (excluding the number combination in which the prime and target was the same number). In each MC block, 20 prime-target combinations were selected, and 70% of the trials were congruent (e.g., the prime was “2” and the target was “1”) and 30% were incongruent (e.g., the prime is “2” and the target was “7”). In each MI block, the other 20 prime-target combinations were selected, and 70% of the trials were incongruent and 30% were congruent. In other words, two different sets of 20 prime-target combinations were selected: one set for the MC blocks and the other set for the MI blocks, without overlap. The 20 prime-target pairs were presented only once per block in a pseudo-randomized order. To construct the MC context, the MC block was presented 12 times, for a total of 12 blocks and 240 trials. The same situation occurred for the MI context. Participants first completed all 12 blocks of one context. After this, they were given short breaks before switching to 12 blocks of the other context. The assignment of the MC and MI contexts was counter-balanced across subjects. Overall, in the prime-target number category task, a specific prime-target digit pair was set to repeat with equal frequency rather than different frequencies under both the MC and MI contexts. All digits appeared as targets and primes in an equal number of trials.

We also took great care to equalize the numerical distance of the targets to the standard 5 because this has been found to affect RTs in this paradigm (Moyer and Landauer, 1967; Koechlin et al., 1999). The distance is the same with congruent and incongruent trials and in both MC and MI contexts.

### Statistical Analysis

The first trial of each block was considered a warm-up trial and removed from further analysis. Extreme RTs (<250 ms or >1000 ms) and erroneous responses were excluded from RT analysis. For each subject, mean RT and overall accuracy were determined for congruent and incongruent conditions. Then, the data were analyzed with separate repeated measures ANOVAs to determine the group effect during two different cognitive control strategies.

In addition to analyses of the mean accuracy and RT, the distributions were analyzed using conditional accuracy functions (CAFs) and delta plots of RT. For this purpose, the RTs of all trials (including both correct and incorrect responses) were rank-ordered and divided into four equal sized bins (bins 1–4) separately for each participant and condition. CAFs were then created to visualize accuracy as a function of RT by plotting accuracy rates against the average RT for each bin. Delta plots were constructed by plotting the average interference effect (i.e., the difference between the incongruent and congruent conditions) as a function of the average RT for each bin.

Response capture (i.e., automatic response activation of the irrelevant dimension of the stimulus) was revealed by CAFs (Wylie et al., 2010) and the strength of the response capture was analyzed by comparing accuracy rates of the fastest RT segment of the CAFs under congruent and incongruent conditions. The strength of response suppression was analyzed by focusing on the slope of the slowest RT segment. The slope value was computed as the difference between the interference effects of Bin 4 and Bin 3 divided by the difference in RT between Bin 4 and Bin 3; more effective inhibition was indicated by more negative delta slopes (Ridderinkhof, 2002a,b; Wylie et al., 2010; Jiang et al., 2012).

## Results

The results are organized as follows. First, we present analyses of the group effects on reactive control processes, including both between-trial and within-trial reactive control. Second, we describe the results of the analyses of group effects on proactive control processes.

### Influence of Aging on the Dynamics of Between-Trial Reactive Control

In order to exclude the contribution of PC effects, we focused our analysis of between-trial reactive control on the MC context trials.

#### Mean RT and Accuracy Effects

Repeated measures ANOVAs on mean RT and mean accuracy were conducted with current trial congruence (congruent vs. incongruent) and previous trial congruence (congruent vs. incongruent) as within-subjects factors and group (older adults vs. younger adults) as a between-subjects factor. In the analysis of RTs, there was a significant interaction between previous congruency, current congruency, and group [*F*(1,58) = 14.78, *p* < 0.05, η^2^ = 0.20]. To analyze this interaction further, separate analyses were conducted for the two groups. For young adults, the current congruency by previous congruency interaction was not significant [*F*(1,29) = 0.03, *p >* 0.05, η^2^ = 0.001], with a 34 ms interference effect for previous congruent trials and a 35 ms interference effect for previous incongruent trials. The interaction of previous congruency and current congruency was significant for older adults [*F*(1,29) = 49.5, *p <* 0.05, η^2^ = 0.63]; the interference effect on RT for previous incongruent trials (82 ms) was significantly larger than that for previous congruent trials (60 ms), revealing a reversed sequential congruence pattern.

In the analysis of accuracy, there was a significant interaction between previous congruency, current congruency, and group [*F*(1,58) = 3.77, *p* = 0.05, η^2^ = 0.06]. To analyze this interaction further, separate analyses were conducted for the two groups. The interaction of previous congruency and current congruency was significant for young adults [*F*(1,29) = 9.02, *p <* 0.05, η^2^ = 0.24], with a larger accuracy difference between congruent and incongruent trials following incongruent trials (6%) compared to that following congruent trials (2%). For older adults, the interaction between current congruency and previous congruency was not significant [*F*(1,29) = 0.38, *p >* 0.05, η^2^ = 0.01]. Therefore, the statistical analyses showed that there was no reliable conflict adaptation in mean RT and accuracy data.

#### The Effect of Between-Trial Reactive Control on Response Capture

In order to explore the effect of between-trial reactive control on response capture, we analyzed the accuracy rate on the first bin of congruent and incongruent trials to isolate these patterns of fast errors. The statistical analysis showed that there was a significant interaction between previous congruency, current congruency, and group [*F*(1,58) = 6.26, *p* < 0.05, η^2^ = 0.09]. To analyze this interaction further, separate analyses were conducted for the two groups. The interaction of previous congruency and current congruency was significant for young adults [*F*(1,29) = 11.10, *p* < 0.05, η^2^ = 0.27]. Further analysis showed a larger accuracy difference between congruent and incongruent trials following incongruent trials (23%) compared to that following congruent trials (16%), revealing a reversed sequential congruence pattern. For older adults, the interaction between current congruency and previous congruency was not significant [*F*(1,29) = 0.005, *p* > 0.05, η^2^ = 0.001].

#### The Effect of Between-Trial Reactive Control on Response Suppression

According to the activation-suppression model, the slope connecting the final two segments of the delta plot is tied to the effectiveness of inhibitory control. Thus, a repeated-measures ANOVA on the slopes of the last delta plot segments was performed with the factors of previous congruency and group. The results showed that there was no significant main effect of previous congruency [*F*(1,58) = 0.07, *p* > 0.05, η^2^ = 0.001], indicating that the slopes of previous incongruent trials were not more negative than were those of previous congruent trials. The interaction between the previous congruency and group was also not significant [*F*(1,58) = 0.07, *p* > 0.05, η^2^ = 0.001]. The same pattern was present in the first and second bins. These results indicated that there was no SC effect on response suppression.

### Influence of Aging on the Dynamics of Within-Trial Reactive Control

To analyze within-trial reactive control such that it was not confounded with between-trial conflict adaption, we focused on current trials preceded by congruent trials in the MC context.

#### Mean RT and Accuracy Rates

**FIGURE 1:**
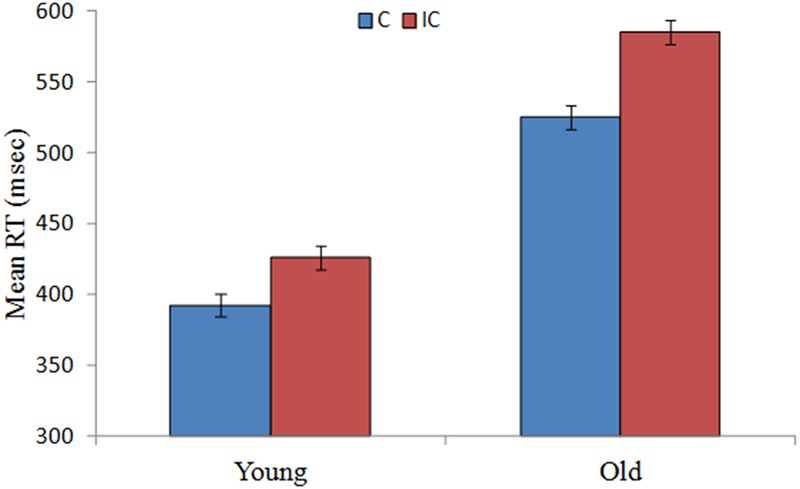
**Mean RTs for young and older adults on congruent (C) and incongruent (IC) trials preceded by congruent trials in the MC context.** Error bars reflect standard errors of the mean.

Repeated-measures ANOVAs on mean RT and mean accuracy were conducted with congruence (congruent vs. incongruent) as a within-subjects factor and group (older adults vs. younger adults) as a between-subjects factor. Older adults were 146 ms slower [*F*(1,58) = 158.28, *p* < 0.001, η^2^ = 0.73] than were younger adults. In addition, RTs were slower [*F*(1,58) = 695.79, *p* < 0.001, η^2^ = 0.92] for incongruent than for congruent trials. The interactions between group and congruence were significant for RT, [*F*(1,58) = 56.67, *p* < 0.001, η^2^ = 0.49], but not accuracy [*F*(1,58) = 0.03, *p* = 0.85, η^2^ = 0.001]. The magnitude of the interference effect on RT for older adults was larger than that for younger adults [*F*(1,58) = 93.79, *p* < 0.001; older: 60 ms; younger: 33 ms]. The mean RTs of older adults and younger adults on current trials preceded by congruent trials in the MC context are presented in **Figure 1**.

#### The Effect of Within-Trial Reactive Control on Response Capture

**FIGURE 2:**
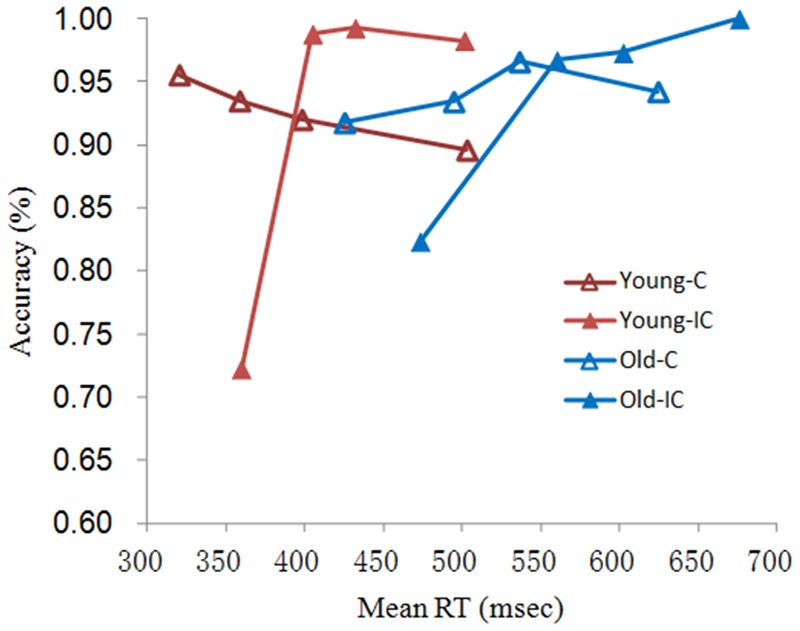
**Conditional accuracy functions for young and older adults on C and IC trial types preceded by corresponding trials in the MC context**.

The CAFs shown in **Figure 2** revealed that most of the errors were fast errors, especially on incongruent trials. Slow responses on incongruent trials as well as both fast and slow responses on congruent trials were associated with higher accuracy. Both older and younger adults showed similar patterns of fast errors, but younger adults made more errors, especially on fast incongruent trials.

To verify these visual impressions, we analyzed the accuracy rate on the first bin of congruent and incongruent trials to isolate these patterns of fast errors. A repeated-measures ANOVA with congruence as a within-subjects factor and group as a between-subjects factor revealed a significant effect of congruence [*F*(1,58) = 50.75, *p* < 0.001, η^2^ = 0.47], indicating that more fast errors occurred on incongruent than on congruent trials. The interaction between group and congruence was significant [*F*(1,58) = 9.13, *p* < 0.001, η^2^ = 0.14], showing that older adults made fewer error responses on incongruent trials than did younger adults. This accords with the activation-suppression model, wherein older adults experience lower levels of initial error response capture from the automatic processing of conflicting interference information.

#### The Effect of Within-Trial Reactive Control on Response Suppression

**FIGURE 3:**
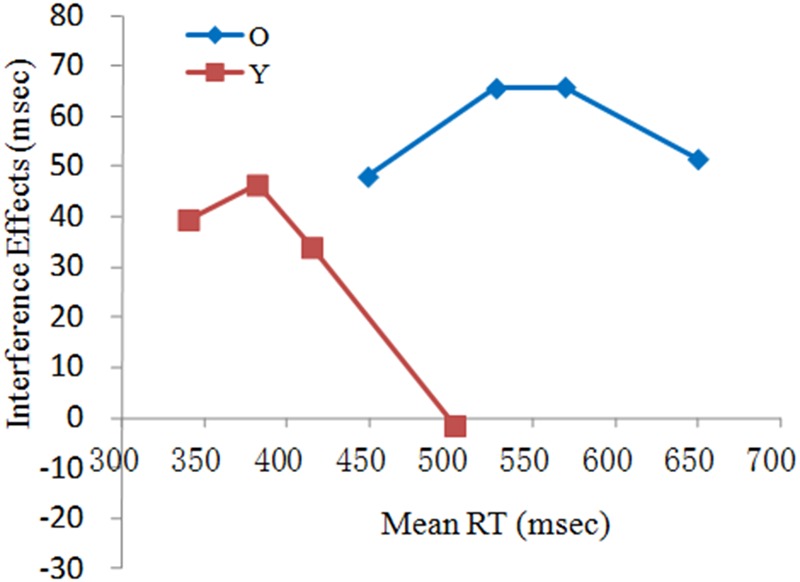
**RT delta plots for young (Y) and older (O) adults on current trials preceded by congruent trials in the MC context**.

The delta plots for older and young adults (**Figure 3**) clearly illustrated the absence of uniformity in the interference effect across the RT distribution. As can be seen in **Figure 3**, the interference effect in younger adults tended to deeply decline with slower RTs, whereas older adults rose progressively in the first two segments and then slightly declined in the last segment. We conducted an ANOVA with group as a between-subjects factor (older, younger) on the final slope of the delta plot to determine any group difference in inhibitory control. The analysis showed that the slope was more negative for younger adults (*M* = -0.46) than for older adults [*M* = -0.21, *F*(1,58) = 7.71, *p* < 0.05, η^2^ = 0.12]. The difference in slope, according to the activation-suppression model, suggested that older adults showed less efficient inhibition than did young adults.

### Influence of Aging on the Dynamics of Proactive Control

#### Mean RT and Accuracy Effects

**FIGURE 4:**
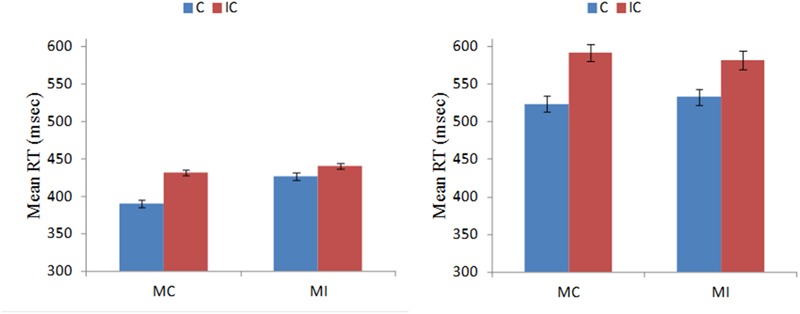
**Mean RTs for young **(Left)** and older **(Right)** adults on the C and IC trial types in the MC and MI contexts.** Error bars reflect the standard error of the means.

An ANOVA with congruence (congruent, incongruent) and proportion congruency (MC, MI) as within-subject factors and group as a between-subject factor showed that RTs were slower [*F*(1,58) = 598.96, *p* < 0.001, η^2^ = 0.91] and accuracy rates were lower [*F*(1,58) = 106.89, *p* < 0.001, η^2^ = 0.65] for incongruent than for congruent trials. The interaction between congruence and proportion congruency on RT [*F*(1,58) = 101.03, *p* < 0.001, η^2^ = 0.64] were also significant. A simple effects analysis of the interaction showed that the interference effect was smaller in the MI context than in the MC context. Of particular importance is our observation that this interaction did not vary by group [*F*(1,58) = 0.21, *p* > 0.05, η^2^ = 0.004]. Specifically, when experiencing more conflict on MI blocks, both older and younger adults were able to adapt to this conflict and minimize it proactively. The mean RTs of both groups for the MC and MI contexts are depicted in **Figure 4**.

#### The Effect of Proactive Control on Response Capture

**FIGURE 5:**
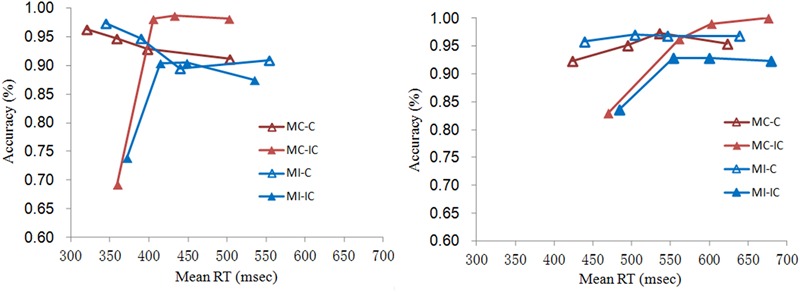
**Conditional accuracy functions for young **(Left)** and older **(Right)** adults on C and IC trial types in the MC and MI contexts**.

**Figure 5** depicts the group CAFs for congruent and incongruent trials in the two contexts. To examine whether the proportion congruency modulated the initial error response, a repeated-measures ANOVA on accuracy rates for the fastest RT bin was conducted with the factors of congruency proportion, congruence, and group. The results showed that more fast errors occurred on incongruent trials [*F*(1,58) = 184.01, *p* < 0.001, η^2^ = 0.76]. Furthermore, the groups differed in the percentage of fast errors [older: 88.7%; younger: 84.2%; *F*(1,58) = 8.68, *p* < 0.01, η^2^ = 0.13]. The analysis also revealed a main effect of proportion congruency [*F*(1,58) = 5.51, *p* < 0.05, η^2^ = 0.09], indicating that the incorrect response capture was lower in the MI context than in the MC context (MC: 85.2%; MI: 87.7%). Most importantly, the main effects of congruency proportion were not modulated by congruency or group, meaning that incorrect response capture was lower in the MI context than in the MC context for not only congruent trials but also incongruent trials, and that this pattern was present for both age groups. Thes e results suggest that the two groups proactively activated control processes to minimize automatic response capture by using information on frequency.

#### The Effect of Proactive Control on Selective Suppression

**FIGURE 6:**
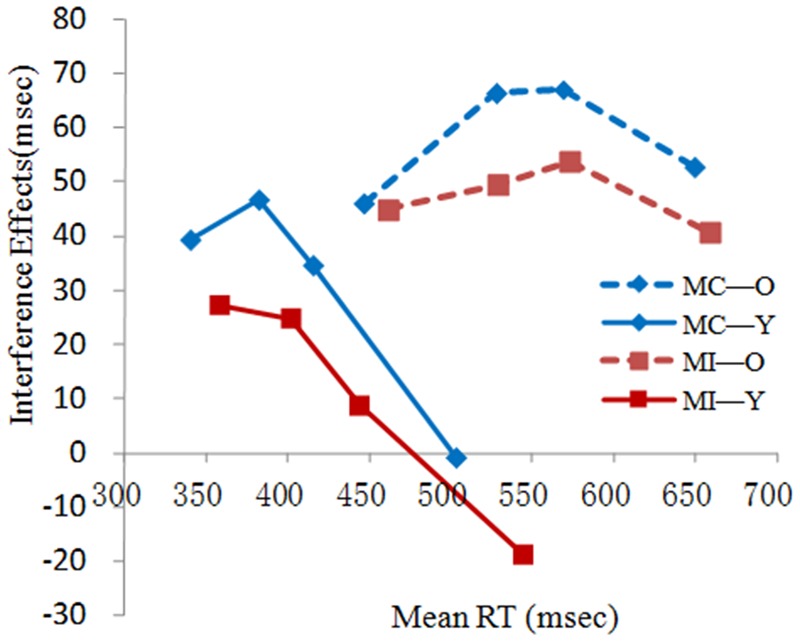
**RT delta plots for young (Y) and older (O) adults in the MC and MI contexts**.

**Figure 6** shows the RT delta plots as a function of group in the two contexts. Repeated measures ANOVAs were performed with the factors of proportion congruency and group on the slopes of the last delta plot segments. The results yielded a significant main effect of group [*F*(1,58) = 9.14, *p* < 0.01, η^2^ = 0.13, Y: -0.34 O: -0.18], indicating that the delta plot slopes for young adults were more negative than were those for older adults. The hypothesized main effect of proportion congruency was not observed, indicating that the slopes in the MI context were not more negative than were those in the MC context. The interaction between the two factors was also non-significant. We then focused on the first and second segments. The ANOVAs showed a main effect of proportion congruency in only the first segment [*F*(1,58) = 5.18, *p* = 0.028, η^2^ = 0.08], indicating that the slopes in the MI context were more negative than were those in the MC context (MI: 0.04; MC: 0.17). This suggested that participants were more likely to suppress the interference information early on in the MI context compared to in the MC context. This pattern was found in both age groups as suggested by the non-significant interaction between proportion congruency and group [*F*(1,58) = 2.40, *p* = 0.13, η^2^ = 0.04]. These results indicated that the two age groups suppress interference information by using information on frequency in the early stage.

## Discussion

The present study investigated the influence of aging on two types of cognitive control under the framework of the dual control mechanism (Braver, 2012). We sought to measure cognitive control in a “pure” context—namely, one in which frequency confounds and stimulus repetitions were controlled for. The performance pattern could thus be explained by pure cognitive control rather than bottom-up processing. In addition, we used the activation-suppression model to investigate the processing dynamics of the two cognitive control functions. This method allowed us to determine whether the groups differed in terms of the strength of incorrect response capture induced by automatic processing or in terms of the efficiency of suppression engaged to reduce the interference. Group effects on reactive and proactive control processes are discussed, respectively, in the following sections.

For reactive control, we differentiated two types: within-trial conflict resolution and between-trial conflict adaption (i.e., SC effects). The statistical analysis showed that there was no reliable SC effect on mean RT and accuracy. Moreover, an analysis of the initial error response capture or late selective suppression also did not reveal any effect of previous trial congruency. The SC effect has been interpreted as reflecting top-down attentional adjustments in cognitive control or, alternatively, bottom-up associative learning such as feature repetitions (Mayr et al., 2003; Hommel et al., 2004; Schmidt and Besner, 2008). Some studies using conflict tasks have demonstrated that SC effects disappear when the stimulus repetition and contingencies learning was either prevented from occurring or removed from analysis after the fact (Nieuwenhuis et al., 2006; Fernandezduque and Knight, 2008; Schmidt and Besner, 2008; Chen and Melara, 2009; Schmidt, 2013). Consistent with these studies, we observed no SC effects in our interference task where feature repetitions were controlled *a priori*.

We then examined the effect of aging on within-trial reactive control. We observed a larger interference effect on RTs in older adults compared with in young adults. A distributional analysis was further used to examine the underlying processing dynamics of these augmented interference effects for older adults in more detail. The analysis of initial accurate rates showed that older adults experienced lower levels of initial error response capture from the automatic processing of conflict information compared to younger adults. This may be explained by the age-related strategy of emphasizing accuracy over speed in older adults (Czernochowski et al., 2010). Next, we considered the effects of aging on the proficiency of response suppression. The results showed that the increase in the interference effect at the slowest RT segment was significantly greater for older adults than for younger adults. This indicated that older adults were less proficient at suppressing the interference arising from incorrect response capture.

The distributional analysis further showed that the decrease in reactive conflict control in older adults stemmed from inefficient suppression, not from stronger incorrect responses. These results further support the hypothesis that a reduction of inhibition efficiency is an important component of age-related cognitive changes (Hasher et al., 2007; Hasher et al., 1991).

With respect to proactive control of interference, we explored whether older adults could represent frequency information and then adapt to it at the list level by comparing the performance of the MI context to that of the MC context. The analyses showed that proportion congruency clearly affected overall RT and accuracy. Specifically, the interference effects dramatically decreased in the MI context, in which incongruent trials were more prevalent. The findings suggested that varying the relative probability of incongruent trials successfully led participants to establish a different task context and then engage in global adjustment in response to proportion information (Gratton et al., 1992; Ridderinkhof, 2002b). Moreover, this effect did not vary with age. The also results indicated that older adults developed a block-wise strategy to control interference proactively, similar to the younger adults (Mutter et al., 2005; Bélanger et al., 2010).

The distribution analysis showed that the modification of behavioral strategies incurred by the relative proportion of incongruent trials was involved in not only reduced initial automatic response activation but also stronger selective suppression. Similarly, the proportion congruency effect did not vary by age. These results suggest that older adults proactively activated control processes to minimize the initial automatic response activation and suppress the interference, similarly to young adults, after frequently experiencing response conflict.

It should be noted that a prominent theory has argued that SC and PC effects might be explained by the same mechanism (Botvinick et al., 2001). Specifically, in the MI context, incongruent trials were more frequent than were congruent trials, which means that incongruent-incongruent (II) transitions were the most common. Thus, there was a reduction in interference effects in the MI context—as in, when there was a high proportion of incongruent trials—as the context had a high proportion of II transitions. Recent studies have questioned this argument, however, by showing behavioral dissociations between PC and SC effects within the context of conflict tasks. Specifically, these studies showed that while SC effects are typically specific to conflict type, PC effects generalize across conflict types (Funes et al., 2010; Torres-Quesada et al., 2013). In the current study, the statistical analysis showed that although significant PC effects were observed, there were no reliable SC effects in either context (i.e., MC or MI). A previous work found similar results. Torres-Quesada et al. (2014) used a paradigm in which two conflict types were randomly intermixed (Simon task and Spatial Stroop task) and the proportion of congruency was manipulated for one conflict type and kept neutral for the other conflict type. The results showed that in conflict-type alternation trials, where SC effects were absent, PC effects were still present. Therefore, the PC effect in the current study did not arise from the accumulation of SC effects. The results indicated that the “proactive control” reflected by PC effects in the present study cannot be explained by the same transient or “reactive control” mechanism proposed to account for SC effects.

The preserved proactive control is inconsistent with the results of previous studies that used the AX-CPT task to examine the aging of cognitive control (Braver et al., 2001; Paxton et al., 2008). These previous studies found that older adults have selective deficits in proactive control. The difference between our study and the two previous studies might be due to the task used. Although closely related, the interference task and AX-CPT tasks might involve some sub-processes affected differentially by age. The dual mechanisms of cognitive control hold that the two types of cognitive control have their own costs and benefits, and that there exists a computational tradeoff between them (Braver and West, 2008; Braver, 2012). There are some situations in which one or the other type of control would be most appropriate, depending on factors such as individual characteristics and task situation. Given that older adults have limited processing resources (Craik and Byrd, 1982; Manard et al., 2014), it may be difficult for them to process the cue proactively and maintain the cue information over the delay in the AX-CPT task (Braver et al., 2001). Thus, older adults prefer to rely predominantly on reactive control mechanisms in the AX-CPT task, as they do not require sustained control over extensive time periods. However, proactive control can be used in a Stroop-like interference task in which the contextual cue is easily available and reliable enough to anticipate upcoming task demands (Braver et al., 2007; Czernochowski et al., 2010). The MI context (in which the incongruent trials were relatively frequent) allowed the participants to anticipate and prevent the inference information. Therefore, in our study, using proactive control might have been relatively easy and a more optimal strategy to use in the MI context, even for older adults. The inconsistency between studies showed that older adults do not use proactive control by default because it is relatively resource demanding. However, it does not mean that older adults show impaired proactive control. This view is supported by evidence showing that older adults shift to a proactive control strategy from a reactive one after task-strategy training in the AX-CPT task (Paxton et al., 2006; Edwards et al., 2010; Braver, 2012). Therefore, it seems that older adults can exhibit the adjustment of proactive control regardless of the study paradigms.

## Conclusion

The present study showed that older adults exhibit a decline in within-trial reactive interference control processes. This decline mainly originated from the relatively lower efficiency of suppression control but not from incorrect response activation, which was consistent with the inhibition deficit hypothesis (Hasher et al., 1991; Hasher and Zacks, 1988). For proactive control, older adults were as effective as younger adults in minimizing automatic response capture and suppressing irrelevant response activation in response to congruency proportion manipulation. These results challenge earlier views that older adults have selective deficits in proactive control but intact reactive control (Braver et al., 2007; Braver, 2012).

## Author Contributions

LX and BW performed the experimental work, analyzed the data, and wrote the manuscript. ZH supervised the project and edited the manuscript. BZ, JJ, and FZ wrote/edited the manuscript.

## Conflict of Interest Statement

The authors declare that the research was conducted in the absence of any commercial or financial relationships that could be construed as a potential conflict of interest.
